# Systemic Inflammation from Periodontal Disease and its Neuropsychiatric Implications

**DOI:** 10.1192/j.eurpsy.2025.1210

**Published:** 2025-08-26

**Authors:** D. Patel, D. Nolasco, B. Carr

**Affiliations:** 1 Medicine, Nova Southeastern University, Davie; 2 Psychiatry, University of Florida, Gainesville, United States

## Abstract

**Introduction:**

Chronic oral health issues, particularly periodontal disease, are significant contributors to systemic inflammation. Emerging evidence indicates that this inflammation can influence neurophysiological processes and contribute to psychiatric disorders, including depression and anxiety. Recent literature review has revealed that periodontal disease and depression share common biomarkers, such as lipopolysaccharide (LPS), C-reactive protein (CRP), interleukin-6 (IL-6), and cortisol.

**Objectives:**

To elucidate the connection between poor oral health and psychiatric disorders through the lens of systemic inflammation and its neurophysiological impacts.

**Methods:**

A comprehensive literature review was conducted using PubMed, PsycINFO, and Google Scholar, focusing on studies from the past decade that examine the relationship between periodontal disease, systemic inflammation, and psychiatric disorders. Key findings regarding the involvement of pro-inflammatory cytokines and their neurophysiological effects were synthesized.

**Results:**

The review highlighted that periodontal disease leads to elevated levels of pro-inflammatory cytokines. Such cytokines as CRP, IL-6, and tumor necrosis factor-alpha (TNF-α) can cross the blood-brain barrier, contributing to neuroinflammation and dysregulation of neurotransmitter systems. One significant effect is on serotonin metabolism, which is critical in the pathophysiology of depression. Pro-inflammatory cytokines can lead to reduced serotonin availability by upregulating genetic expression of serotonin transporters and increased serotonin metabolism. Given the interplay between inflammatory cytokines and neurotransmitter metabolism, the studies indicate that individuals with periodontal disease are more likely to exhibit depressive and anxiety symptoms, with neuroinflammation serving as a mediating factor.

**Table 1: Impact of Pro-inflammatory Cytokines on Neurophysiological Processes**

**Table:** 

Cytokine	Effect on Neurophysiology
CRP	Contributes to neuroinflammation and affects processing skills, motor function, and working memory.
IL-6	Promotes neuroinflammatory responses via impacting neurotransmitter regulation and gut-microbiota-brain axis
TNF-α	Crosses BBB, leading to neuroinflammation and activates hypothalamus-pituitary axis leading to serotonin and tryptophan dysregulation.

**Conclusions:**

Systemic inflammation resulting from poor oral health significantly impacts neurophysiological processes associated with psychiatric disorders. These findings underscore the need for integrated healthcare approaches that consider both oral and mental health to mitigate the adverse effects of neuroinflammation on psychiatric well-being.

**Disclosure of Interest:**

None Declared

